# Efficient Fluoride Removal from Aqueous Solution Using Zirconium-Based Composite Nanofiber Membranes

**DOI:** 10.3390/membranes11020147

**Published:** 2021-02-20

**Authors:** Alaa Mohamed, Elvia P. Valadez Sanchez, Evgenia Bogdanova, Britta Bergfeldt, Ammar Mahmood, Roman V. Ostvald, Tawheed Hashem

**Affiliations:** 1 Institute of Functional Interfaces (IFG), Karlsruhe Institute of Technology (KIT), Hermann-von Helmholtz-Platz 1, 76344 Eggenstein-Leopoldshafen, Germany; valadez.elvia@gmail.com (E.P.V.S.); ammar.mahmood@student.kit.edu (A.M.); 2 Egypt Nanotechnology Center, EGNC, Cairo University, Giza 12613, Egypt; 3 School of Nuclear Science and Engineering, National Research Tomsk Polytechnic University, 634050 Tomsk, Russia; evgeniyabog@mail.ru (E.B.); ostvald@tpu.ru (R.V.O.); 4 Institute for Technical Chemistry (ITC), Karlsruhe Institute for Technology (KIT), Hermann-von Helmholtz-Platz 1, 76344 Eggenstein-Leopoldshafen, Germany; Britta.Bergfeldt@kit.edu; 5 International X-ray Optics Lab, Institute of Physics and Technology, National Research Tomsk Polytechnic University (TPU), 30 Lenin Ave., 634050 Tomsk, Russia

**Keywords:** metal-organic framework, UiO-66, UiO-66-NH_2_, water treatment, adsorption mechanisms

## Abstract

Herein, composite nanofiber membranes (CNMs) derived from UiO-66 and UiO-66-NH_2_ Zr-metal-organic frameworks (MOFs) were successfully prepared, and they exhibited high performance in adsorptive fluoride removal from aqueous media. The resultant CNMs were confirmed using different techniques, such as X-ray diffraction (XRD), field emission scanning electron microscopy (FE-SEM), and Brunauer–Emmett–Teller (BET) in addition to Fourier-transform infrared spectroscopy (FTIR). The parameters that govern the fluoride adsorption were evaluated, including adsorbent dose, contact time, and pH value, in addition to initial concentration. The crystalline structures of CNMs exhibited high hydrothermal stability and remained intact after fluoride adsorption. It could also be observed that the adsorbent dose has a significant effect on fluoride removal at high alkaline values. The results show that UiO-66-NH_2_ CNM exhibited high fluoride removal due to electrostatic interactions that strongly existed between F^−^ and metal sites in MOF in addition to hydrogen bonds formed with MOF amino groups. The fluoride removal efficiency reached 95% under optimal conditions of 20 mg L^−1^, pH of 8, and 40% adsorbent dose at 60 min. The results revealed that UiO-66-NH_2_ CNM possesses a high maximum adsorption capacity (95 mg L^−1^) over UiO-66 CNM (75 mg L^−1^), which exhibited better fitting with the pseudo-second-order model. Moreover, when the initial fluoride concentration increased from 20 to 100 mg/L, fluoride adsorption decreased by 57% (UiO-66 CNM) and 30% (UiO-66-NH_2_ CNM) after 60 min. After three cycles, CNM revealed the regeneration ability, demonstrating that UiO-66-NH_2_ CNMs are auspicious adsorbents for fluoride from an aqueous medium.

## 1. Introduction

Drinking water treatment poses a challenge attributed to industrial wastewater’s daily water resources pollution [1,2]. Fluoride is one of the major pollutants that harmfully affects life, especially human beings [3,4]. Typically, it exhibits essential contribution to bone function and prevents dental caries [5,6]. Nevertheless, according to World Health Organization (WHO), fluoride concentration must be <1.5 mg/L or else it could result in severe illness in humans such as molting of teeth; reduced IQ; and long-term damage to brain, liver, and kidneys as well as other organs [7,8]. Fluoride enters water bodies by the weathering process of minerals rich in fluoride and as a result of anthropogenic activities such as industrial drains [9,10]. Fluoride-containing water bodies is a critical problem for tropical nations such as India, Sri Lanka, as well as various countries in Africa. An efficient approach to overcome this issue is de-fluoridation, which can be performed using ion-exchange processes, dialysis, adsorption, and membrane-based processes [11,12,13].

The adsorption process is mostly used because of its simplicity and availability of different adsorbents types [14,15]. Overall, adsorption on a solid surface demonstrates flexibility, simplicity, and suitability to treat drinking water [16,17]. Such a process is also effective and works over an extensive range of pH values and lower residual concentrations in comparison to other methods [18]. For these reasons, a list of adsorbents was investigated to evaluate their possible use as de-fluoridating materials, including activated alumina, activated alumina coated silica gel, bone charcoal, tri-calcium phosphate, activated carbon, activated soil sorbent, calcite, activated coconut shell powder, activated sawdust, groundnut shell, serpentine, coffee husk, activated fly ash, metal-organic frameworks (MOFs), rice husk, magnesia, defluoron-1, defluoron-2, and so on [19,20,21,22,23,24]. In general, adsorptive fluoride removal can reach up to 90%, proving that adsorption is a highly efficient technique in this regard in addition to its cost-effectiveness and simplicity. However, to meet the requirements of potable water, high-capacity and selective adsorbents must be developed.

MOFs manifest great interest in many fields because their porous structures originate from metal clusters and organic ligands, leading to diverse organic–inorganic hybrid linkages [25,26]. MOFs have been investigated for various purposes such as removing toxic gases, hydrogen storage, and CO_2_ adsorption [27,28,29]. Furthermore, many MOF types have shown high capacities for removing pollutants from water, particularly for de-fluoridation processes [30,31]. In this context, the zirconium-based MOF UiO-66 displays a relatively higher adsorption capacity towards adsorptive fluoride removal than Cr-, Fe-, Al-, and Hf-MOFs [32,33]. However, the equilibrium isotherm, stability, kinetics, and thermodynamics regarding UiO-66 use in de-fluoridation of water have not been reported. Furthermore, UiO-66-NH_2_ facilitates fluoride adsorption through electrostatic attraction and hydrogen bonding originated by amino groups. 

Membrane separation processes are preferred by the industry for de-fluoridation of groundwater, wastewater, and seawater [34,35,36]. For membrane-based separation, a unique semipermeable membrane can separate particles based on their molecular shape and size. This membrane can be a typical thin, nonporous, or porous film composed of a metallic or ceramic material or even a gas or liquid [37,38]. One important feature is that the membrane should remain intact without dissolution or cracking in the given medium [39,40]. Typical membrane-based separations for removing fluoride ions include reverse osmosis, nanofiltration, and electrodialysis. Therefore, the combination of highly efficient adsorbent materials with membrane technologies can improve water treatment adsorption performance [41,42]. Considering these points, we herein investigated the preparation of composite nanofiber membranes (CNMs) based on UiO-66 and its amino version (UiO-66-NH_2_).

Electrospinning is a resourceful technique for creating fibrous scaffolds suitable for a wide variety of nanotechnology applications [43,44]. Electrospun CNMs were produced containing zirconium-based water-stable MOF particles supported on polyacrylonitrile (PAN) nanofibers prepared by co-electrospinning. After the dispersion of MOF particles in an organic polymer, their existence was confirmed using X-ray diffraction (XRD), scanning electron microscopy (SEM), and Fourier transform infrared (FTIR) spectroscopy. Besides, XRD was utilized for studying variations in their structures in terms of crystallinity and stability before and after fluoride ions adsorption. Pristine zirconium MOF adsorbents (UiO-66 and UiO-66-NH_2_), in addition to their corresponding CNMs, were investigated for fluoride adsorption. Numerous parameters were explored for adsorption process, such as adsorbent dose, concentration of fluoride ions, contact time, and pH value. 

## 2. Materials and Methods

### 2.1. Materials

Zirconium (IV) chloride (ZrCl_4_), 1,4-benzenedicarboxylic acid (H_2_BDC), 2-aminoterephthalic acid, and hydrochloric acid (37%) were obtained from Merck Company, Germany. Polyvinylidene fluoride (PVDF), sodium fluoride, and *N*,*N*′-dimethylformamide (DMF) were supplied by Sigma-Aldrich, Darmstadt, Germany. 

### 2.2. Preparation of UiO-66 and UiO-66-NH_2_ Nanofibers

Through a solvothermal method, UiO-66 as well as UiO-66-NH2 powders were formed according to our previous studies. Briefly, a mixture of ZrCl_4_ (1.68 g), H_2_BDC (0.96 g), DMF (40 mL), and 4 mL of 37% HCl were heated at 80 °C for 8 h to afford UiO-66. Following reaction completion, mixture was centrifuged, followed by frequent washing using DMF and twice with ethanol to eliminate residual DMF. For UiO-66-NH_2_ synthesis, ZrCl_4_ (0.63 g) as well as 2-aminoterephthalic acid (0.68 g) were dissolved in DMF and then 1 mL of 37% HCl was added to obtain more crystals. After heating at 80 °C with stirring for 7 h, followed by cooling, the collected product was washed using DMF and ethanol (twice), and then dried in the oven overnight. 

For preparing nanofibers derived from UiO-66 and UiO-66-NH_2_, PVDF and different loadings of MOF powders (10, 20, and 40 wt.%) were dissolved in DMF, followed by stirring at 40 °C for 6 h. The CNMs were electrospun on a flat aluminum foil at 0.3 mL h^−1^ flow rate 15 kV, and 20 cm distance between the needle and collector. After electrospinning process completion, the composites were left to dry at 60 °C in an oven for 3 h to remove residual solvent.

### 2.3. Fluoride Adsorption with UiO-66-NH_2_

Batch adsorptive removal experiments were used to investigate the adsorption capacity of composite nanofibers. Such experiments were performed in a glass vial (100 mL) charged with CNM (20 mg) and 20 mg/L concentration of fluoride ions (100 mL). After that, the vial was shaken for different periods at 200 rpm. After adsorption experiments at specified intervals, the solutions were separated and fluoride concentration was determined by ion chromatography (IC, Thermo Fisher Scientific GmbH, Germany). In this study, all the experiments were performed three times to ensure consistency and reproducibility of the results and the average value was recorded. The adsorption capacity and adsorption removal were determined based on the following equations (Equations (1) and (2)):

(1) $$\mathrm{Adsorption}\;\mathrm{capacity}\;(q_{e})\;=\;\frac{(C_{o}-C_{t})V}{m}$$

(2) $$\mathrm{Adsorption}\;\mathrm{removal}\;(\%)\;=\;\frac{C_{o}-C_{t}}{C_{i}}\cdot 100$$

where *C_o_* and *C_t_* (mg L^−1^) refer to initial as well as equilibrium fluoride concentrations, *m* (g) refers to CNM weight, and *V* refers to total solution volume (L). The pH influence was examined using different prepared solutions with a pH range of 2–12 by means of 0.1 M NaOH and HCl. Considering that stability and reusability of adsorbents are important criteria for practical implementation towards environmental contaminants’ degradation, all adsorbents were cleaned, dried, regenerated, and reused for several cycles.

### 2.4. Characterization

The prepared composite nanofiber membranes were characterized before and after fluoride adsorption experiments with field emission scanning electron microscopy (FE-SEM, FEI Philips XL 30) and X-ray diffraction (XRD, D8-Advance, Bruker, Billerica, MA, USA) at 12 kV in the range of 2θ from 3° to 30°. An ATR-FTIR spectrophotometer was utilized to obtain FTIR spectral data of the PVDF nanofiber, UiO-66, and UiO-66-NH_2_ in addition to composite nanofiber membranes before and after adsorption. N_2_ physisorption measurements at 77 K were used to determine the Brunauer–Emmett–Teller (BET) surface area of each sample. The spectra were obtained in the range of 4000–400 cm^−1^. Fluoride detection was analyzed using an ion chromatography system from Thermo Fisher Scientific GmbH Germany (ICS 2000).

### 2.5. Kinetics Study

Identifying the adsorption kinetics and mechanisms of fluoride can be achieved using two models that include pseudo-first-order as well as pseudo-second-order models. These models are expressed as detailed in Table 1.

## 3. Results and Discussion

### 3.1. Characteristics of Adsorbents

**Figure 1:**
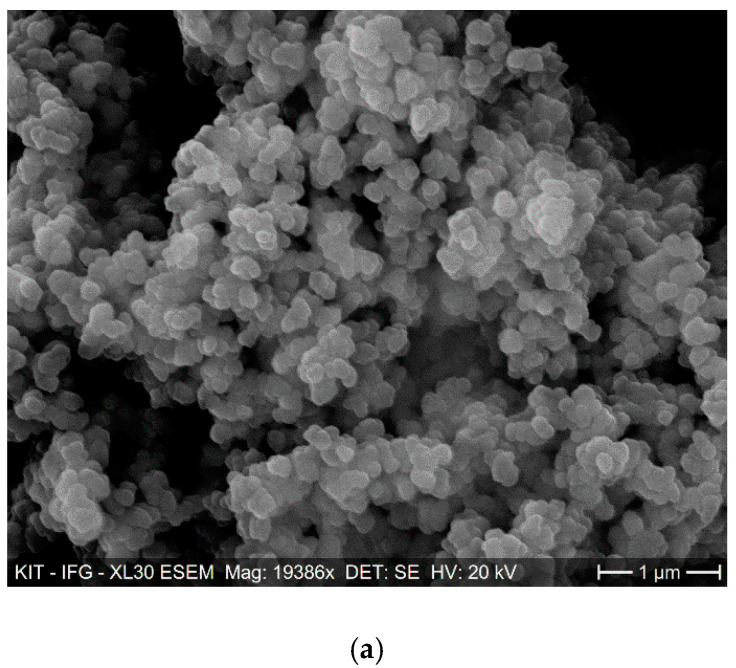

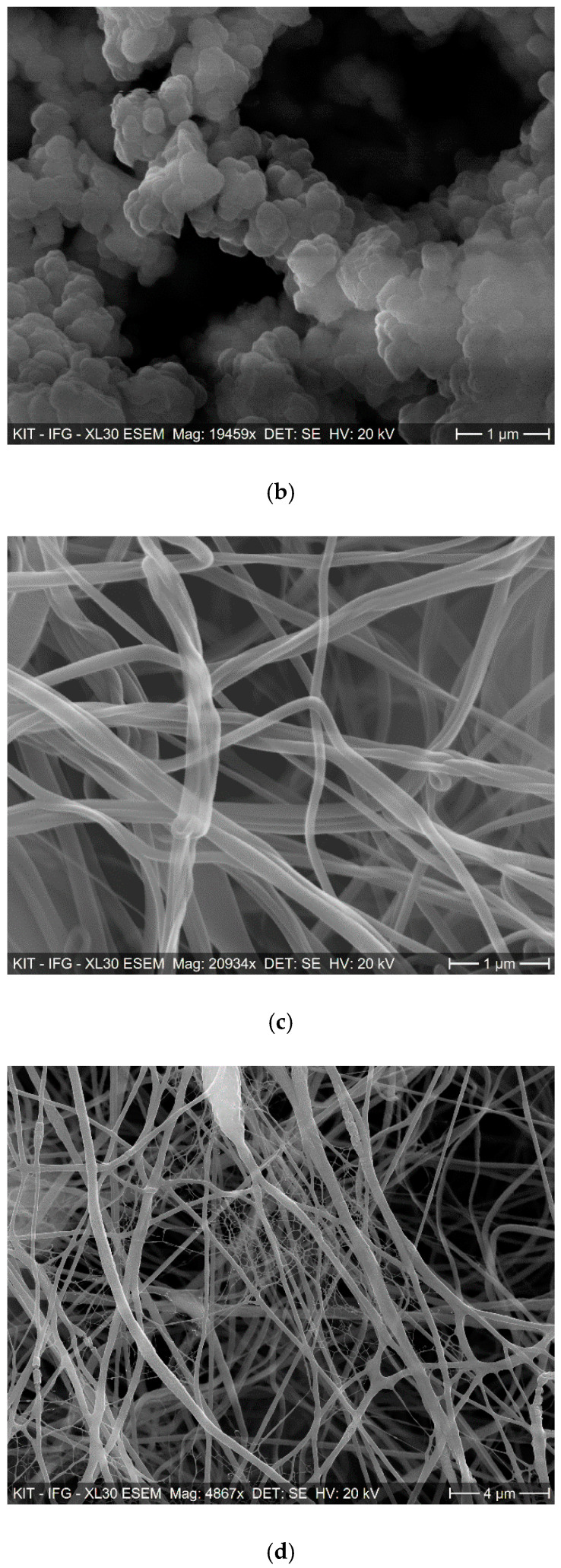

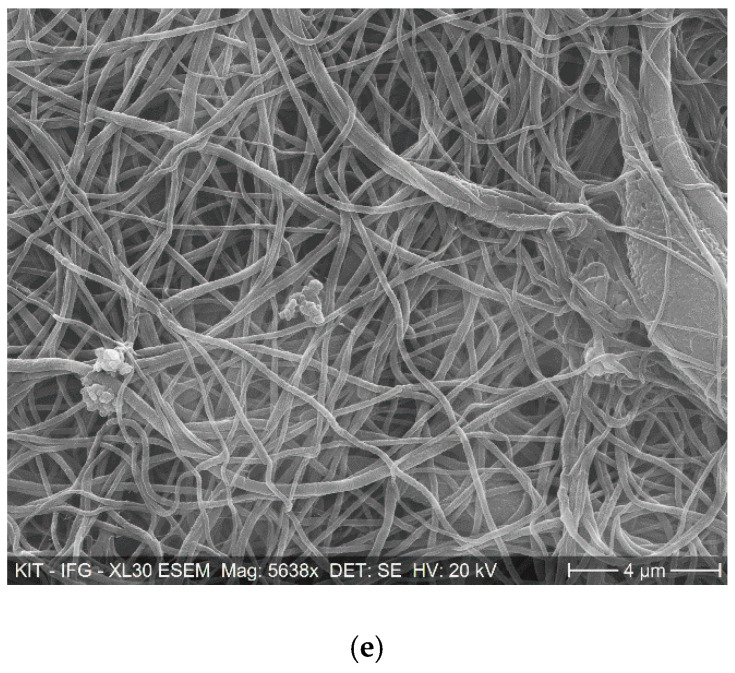
FE-SEM images of (**a**) UiO-66, (**b**) UiO-66-NH_2_, (**c**) polyvinylidene fluoride (PVDF) nanofibers, (**d**) pristine UiO-66-NH_2_ composite nanofiber membrane (CNM), and (**e**) UiO-66-NH_2_ CNM following the adsorption process.

Figure 1 displays images of FE-SEM for zirconium MOFs (UiO-66 as well as UiO-66-NH_2_), PVDF nanofiber, and the composite nanofiber membrane prior to and following adsorption. The morphology of the synthesized UiO-66 and UiO-66-NH_2_ have octahedral crystals with an average size of 100 ± 15 nm and 250 ± 30 nm, respectively. In comparison, the morphology of the CNMs have smooth and uniform structures with an average size of 110 ± 10 nm. Moreover, Figure 1e demonstrates CNM stability after adsorption experiments, revealing no changes in morphology. In addition, the BET surface area of the UiO-66, UiO-66-NH_2_ powder, and the CNM are 1450, 1245, and 425 m^2^ g^−1^, respectively.

**Figure 2:**
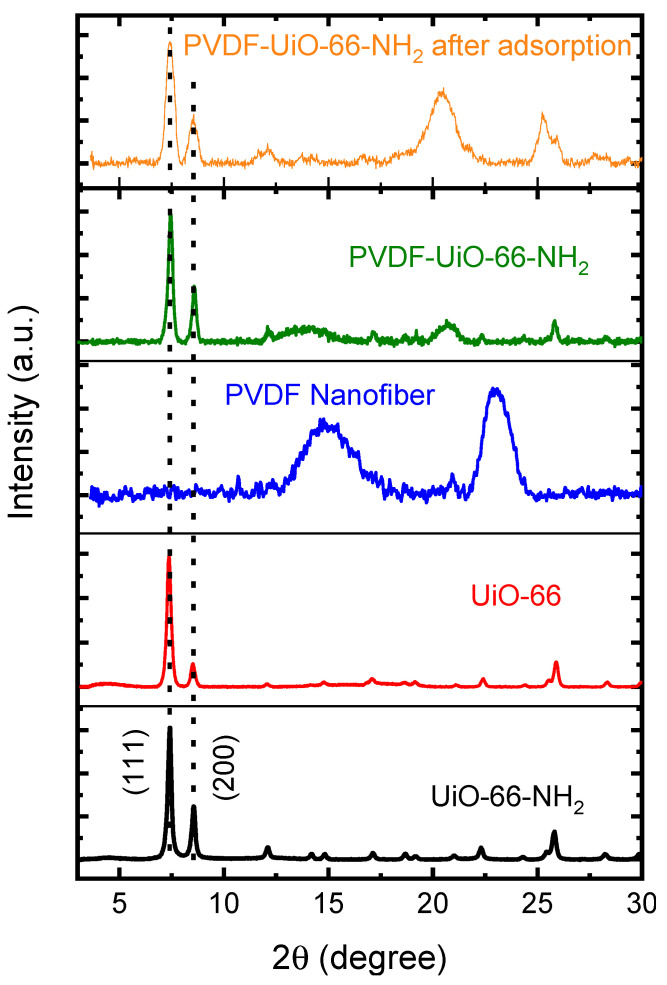
Patterns of XRD for UiO-66-NH_2_, UiO-66, PVDF nanofiber, and UiO-66-NH_2_ CNM prior to and following adsorption at pH 12.

Figure 2 manifests patterns of XRD for UiO-66-NH_2_, UiO-66, the PVDF nanofiber, and the UiO-66-NH_2_ composite nanofiber membrane prior to and following fluoride adsorption. The patterns supported that UiO-66-NH_2_ MOF was successfully incorporated and stabilized into the PVDF nanofiber following fluoride adsorption. The structure of the composite nanofiber membrane remained intact, crystalline, and hydrothermally stable after the fluoride adsorption experiments. The two peaks at 2θ = 7° and 8.45° represent the UiO-66-NH_2_ structure compared to primitive UiO-66-NH_2_, which exhibits excellent agreement with literature reports [45,46].

**Figure 3:**
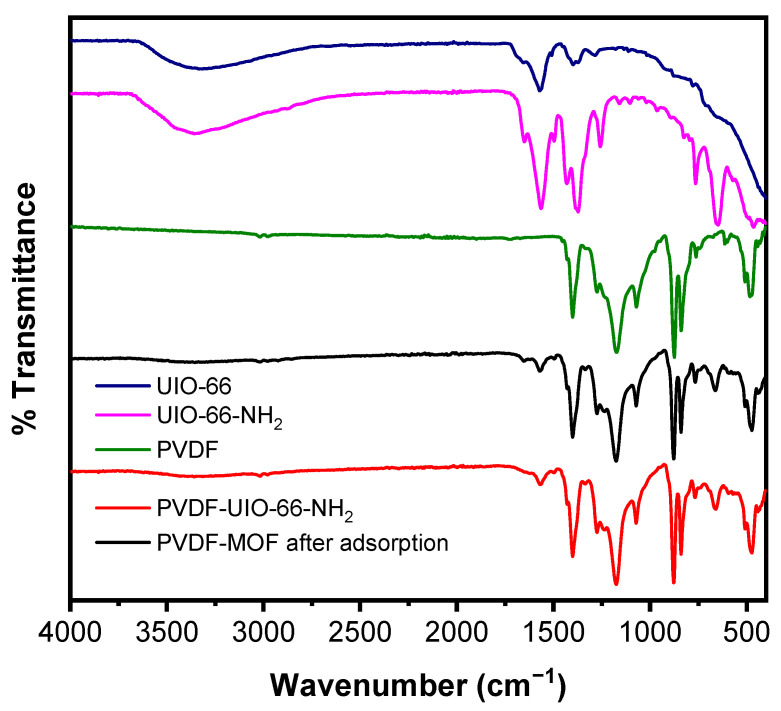
FTIR spectra of UiO-66, UiO-66-NH_2_, PVDF nanofibers, and UiO-66-NH_2_ CNM before and after adsorption.

Figure 3 presents FTIR spectral data for UiO-66 and UiO-66-NH_2_ MOFs, PVDF nanofibers, and UiO-66-NH_2_ CNM prior to and following adsorption, confirming CNM chemical composition. The band identified the amine groups (–NH2) at 3332 cm^−1^, C–O bonds in carboxylate groups were identified by bands at 1372 and 1564 cm^−1^, while 1427 cm^−1^ band was assigned to C–C vibrational bond [47,48]. The broad band at 3346 cm^−1^ was assigned to Zr–OH bonds as well as N–H bonds (asymmetric and symmetric stretching) [49]. For Zr–O and C–N stretching, they exhibited absorptions at 662 and 765 cm^−1^ in addition to 1258 and 1339 cm^−1^, respectively [50]. The spectra confirmed the unchanged composition of CNM before and after adsorption experiments.

### 3.2. FA Adsorption Performance

**Figure 4:**
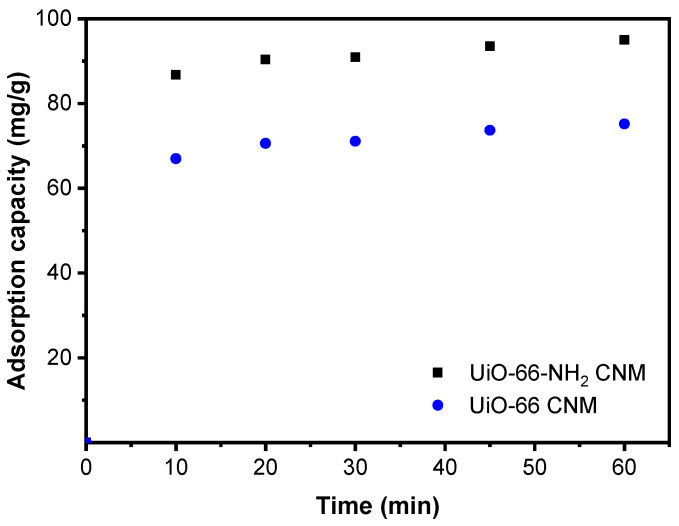
Fluoride adsorption capacity of CNMs as a function of contact time.

Composite nanofiber membranes derived from UiO-66 and its amino version exhibited adsorptive removal performance for fluoride ions, as presented in Figure 4. For CNMs based on UiO-66 and UiO-66-NH_2_, they respectively revealed maximum adsorption capacities of 75 and 95 mg g^−1^. Specifically for UiO-66-NH_2_ CNM, the increased adsorption capacity is because of amino groups, where the metal site can interact through electrostatic interactions with negatively charged sites with the formation of hydrogen bonds [46].

### 3.3. Effect of Adsorbent Dose, Contact Time, pH Value, and Initial Concentration on Removal Efficiency of UiO-66 and UiO-66-NH_2_ CNMs

**Figure 5:**
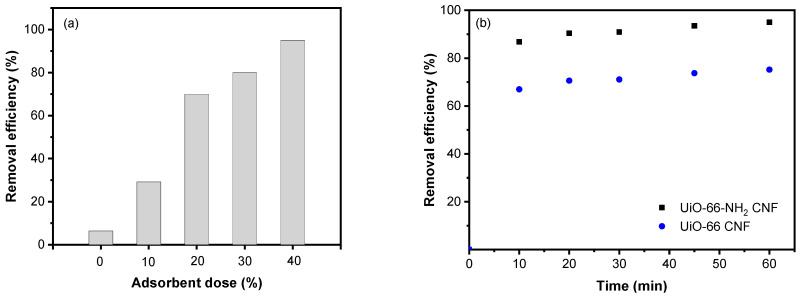

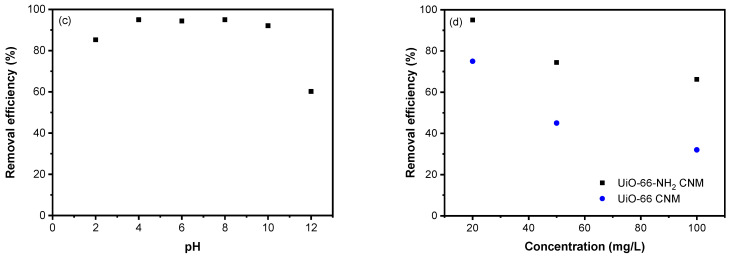
Effect of (**a**) adsorbent dose, (**b**) contact time, (**c**) pH, and (**d**) concentration on the removal efficiency of UiO-66-NH_2_.

For an effective adsorption process, the adsorbent dose is a significant parameter that must be considered [51]. A series of experiments were conducted using different concentrations (10–40 wt.%) of UiO-66-NH_2_ in CNM at a fluoride concentration of 20 mg/L, 60 min, and a pH of 8. The results presented in Figure 5a demonstrate that adsorption performance upsurged rapidly when the UiO-66-NH_2_ amount was increased. This behavior results from the increased accessible active centers located on CNM. Figure 5b displays the contact time influence on removing fluoride ions using a 40 wt.% adsorbent dose when CNM derived from UiO-66 and its amino version were utilized. According to the results, UiO-66-NH_2_ CNM showed 95% maximum removal efficiency whereas UiO-66 CNM had only 75% removal efficiency.

Furthermore, the pH of the medium is another major parameter influencing the adsorption capacity of the adsorbent since it affects the adsorbent surface charge [52]. Figure 5c shows CNM efficiency in fluoride removal at different pH values. These data determined that fluoride removal is not changed substantially except for at a pH of 12. Optimal fluoride removal for UiO-66-NH_2_ CNM was then suggested within a pH range of 4–10, indicating that UiO-66-NH_2_ demonstrates stability under neutral and acidic conditions. When the pH was changed to 12, fluoride removal was reduced suddenly by about 37%, consistent with preceding studies using high pH values [53]. This behavior could be due to the negative adsorbent surface and competition between fluoride and OH^-^ groups, which reduce the adsorption capacity [54,55]. As discussed in previous studies, there is also a stability issue of UiO-66-NH2 under alkaline conditions [56]. Hence, after adsorption at pH 12, the XRD of CNM was measured, as shown in Figure 2. It can be observed that there is no change in CNM structure, indicating that crystallinity remained intact. Therefore, the UiO-66-NH_2_ composite nanofiber membrane demonstrated high stability and adsorption removal over a wide range of pH.

Additionally, various fluoride concentrations were tested using UiO-66 and UiO-66-NH_2_ CNMs (Figure 5d). When the initial fluoride concentration increased from 20 to 100 mg/L, fluoride adsorption decreased by 57% (UiO-66 CNM) and 30% (UiO-66-NH_2_ CNM) after 60 min. This behavior might result from the saturation of adsorbent and occupation of adsorption sites into composite nanofiber surfaces at high concentrations [57]. The results of the current study were compared with those of previous studies to investigate the removal performance of the prepared CNM as summarized in Table 2.

### 3.4. Mechanism

The mechanism of fluoride adsorption may be attributed to metal centers exposed in UiO-66 and UiO-66-NH_2_ MOFs. Due to unsaturated coordination on MOFs’ metal centers, they can exhibit partial positive charges [48,58], resulting in electrostatic interactions with negatively charged sites. Moreover, UiO-66-NH_2_ contains amino groups, which produce hydrogen bonds with fluoride. Therefore, amine groups’ existence can improve fluoride adsorption consistent with the observed higher adsorption capacity held by UiO-66-NH2 compared to UiO-66. Furthermore, the adsorption mechanism can also be due to hydroxyl sites shown previously in FTIR results, where increasing the number of –OH groups improves the fluoride adsorption [59]. Finally, electrostatic and hydrogen-bond interactions were considered the primary adsorption mechanisms in this study.

### 3.5. Regeneration Studies

**Figure 6:**
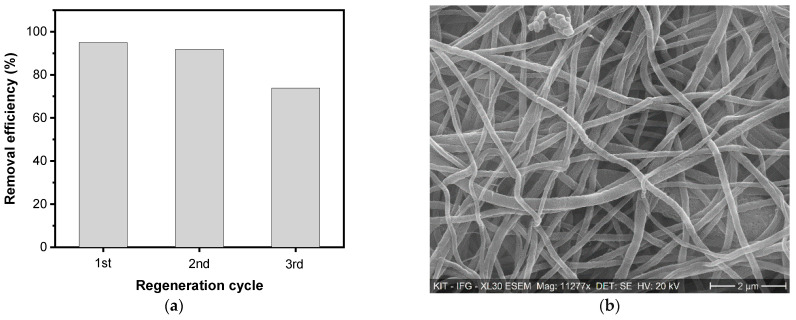
(**a**) Regeneration studies and (**b**) SEM image of CNM after various cycles (20 mg L^−1^, pH of 8, and at 60 min).

Adsorbent reusability is a significant element considered in commercial applications. The composite nanofiber membrane was explored for three recycling experiments as shown in Figure 6. Following each cycle, the membranes were carefully washed using deionized (DI) water, followed by ethanol, and dried in the oven before the next cycle with fresh fluoride. The results showed that the removal efficiencies between the first and second cycles remain almost unchanged. The removal efficiency between the second and third cycles were then lowered to 74%.

## 4. Conclusions

We herein investigated composite nanofiber membranes originating from UiO-66 and UiO-66-NH_2_ Zr-MOFs towards fluoride adsorption. The results showed a successful synthesis of MOF powders and composite nanofiber membranes; their stability after fluoride adsorption experiments was confirmed with no crystallinity, morphology, or composition changes. The results also demonstrated that UiO-66-NH_2_ CNM exhibits adsorption capacities (95 mg g^−1^) higher than UiO-66 CNM (75 mg g^−1^) as a result of hydrogen bonds formation and strong electrostatic interactions between fluoride and UiO-66-NH_2_ CNM. The adsorptive fluoride removal depends on UiO-66-NH_2_ dose, contact time, pH values, and initial fluoride concentration. UiO-66-NH_2_ CNM shows high removal performance under acidic and neutral conditions. Overall, the UiO-66-NH_2_ composite nanofiber membrane appears to be a promising alternative that can be studied more deeply for industrial wastewater and water treatment.

## Figures and Tables

**Table 1:** Kinetic equations of linear and nonlinear pseudo-first-order as well as pseudo-second-order models.

Kinetic Models	Linear Equation	Non-Linear Equation
Pseudo-first-order	$\log(q_{e}-q_{t})=\log\;q_{e\;}-\frac{k_{1}}{2.303}t$	$q_{t}=q_{e\;}(1-e^{-k_{3}t})$
Pseudo-second-order	$\frac{t}{q_{t}}=\frac{1}{k_{2}q_{e}^{2}}+\frac{1}{q_{e}}t$	$q_{t}=\frac{k_{4}q_{e}{}^{2}t}{1+k_{4}q_{e}t}$

*q_e_* and *q_t_*, respectively, refer to fluoride adsorption capacities (mg/g) determined at equilibrium and time (t); *k*_1_ and *k*_2_ refer to the rate constants of the linear first-order and second-order models, respectively; and *k*_3_ and *k*_4_ refer to the rate constants of nonlinear first-order (1 min^−1^) and second-order (g/mg min) adsorption.

**Table 2:** Comparison of the fluoride removal performance with other reported adsorbents.

Material	Concentration (mg L^−1^)	Removal Efficiency (%)	Time (min)	Reference
Alumina-zeolite	4.83	100	20	[19]
MOF-801	10	97	40	[20]
GO/Alumina	20	100	90	[21]
UiO-66-NH_2_	20	100	30	[48]
UiO-66	10	100	80	[53]
Granular ferric hydroxide	10	95	300	[54]
UiO-66 composite nanofibers	20	70	20	**This work**
UiO-66-NH_2_ composite nanofibers	20	97	20	**This work**

MOF: metal-organic framework; GO: Graphene oxide; UiO: Universitetet i Oslo.

## Data Availability

Not applicable.
